# Italian adaptation of the Uniform Data Set Neuropsychological Test Battery (I-UDSNB 1.0): development and normative data

**DOI:** 10.1186/s13195-022-01056-x

**Published:** 2022-08-19

**Authors:** Francesca Conca, Valentina Esposito, Francesco Rundo, Davide Quaranta, Cristina Muscio, Rosa Manenti, Giulia Caruso, Ugo Lucca, Alessia Antonella Galbussera, Sonia Di Tella, Francesca Baglio, Federica L’Abbate, Elisa Canu, Valentina Catania, Massimo Filippi, Giulia Mattavelli, Barbara Poletti, Vincenzo Silani, Raffaele Lodi, Maddalena De Matteis, Michelangelo Stanzani Maserati, Andrea Arighi, Emanuela Rotondo, Antonio Tanzilli, Andrea Pace, Federica Garramone, Carlo Cavaliere, Matteo Pardini, Cristiano Rizzetto, Sandro Sorbi, Roberta Perri, Pietro Tiraboschi, Nicola Canessa, Maria Cotelli, Raffaele Ferri, Sandra Weintraub, Camillo Marra, Fabrizio Tagliavini, Eleonora Catricalà, Stefano Francesco Cappa

**Affiliations:** 1 IRCCS Mondino Foundation, Pavia, Italy; 2 Department of Neurology IC, Oasi Research Institute – IRCCS, Troina, Italy; 3 Neurology Unit, Fondazione Policlinico Universitario “A. Gemelli” IRCCS, Rome, Italy; 4 Department of Psychology, Catholic University of the Sacred Heart, Milan, Italy; 5 Present address: ASST Bergamo Ovest, Treviglio, Italy; 6 Fondazione IRCCS Istituto Neurologico Carlo Besta, Milan, Italy; 7 IRCCS Istituto Centro San Giovanni di Dio Fatebenefratelli, Brescia, Italy; 8 Laboratory of Clinical and Behavioural Neurology, IRCCS Santa Lucia Foundation, Rome, Italy; 9 Laboratory of Geriatric Neuropsychiatry, Department of Neuroscience, Istituto di Ricerche Farmacologiche Mario Negri IRCCS, Milan, Italy; 10 IRCCS Fondazione Don Carlo Gnocchi, ONLUS, Milan, Italy; 11 Neuroimaging Research Unit, Division of Neuroscience, IRCCS San Raffaele Scientific Institute, Milan, Italy; 12 Unit of Psychology I.C., Oasi Research Institute–IRCCS, Troina, Italy; 13 Neurology Unit, Neurorehabilitation Unit, and Neurophysiology Service, IRCCS San Raffaele Scientific Institute, Vita-Salute San Raffaele University, Milan, Italy; 14 IUSS Cognitive Neuroscience (ICON) Center, Scuola Universitaria Superiore IUSS, Palazzo del Broletto, Piazza Vittoria 15, 27100 Pavia, Italy; 15 Istituti Clinici Scientifici Maugeri IRCCS, Cognitive Neuroscience Laboratory of Pavia Institute, Pavia, Italy; 16 Department of Neurology and Laboratory of Neuroscience, IRCCS Istituto Auxologico Italiano, Milan, Italy; 17 Aldo Ravelli Research Center for Neurotechnology and Experimental Brain Therapeutics, Università degli studi di Milano, Milan, Italy; 18 IRCCS Istituto delle Scienze Neurologiche di Bologna, Bologna, Italy; 19 Fondazione IRCSS ca’ Granda, Ospedale Policlinico, Milan, Italy; 20 Neuro-Oncology Unit, IRCCS Regina Elena National Cancer Institute, Rome, Italy; 21 IRCCS Synlab SDN of Naples, Naples, Italy; 22 IRCCS Ospedale Policlinico San Martino, Genoa, Italy; 23 Department of Neuroscience (DINOGMI), University of Genoa, Genoa, Italy; 24 Mesulam Center for Cognitive Neurology and Alzheimer’s Disease and Department of Psychiatry and Behavioral Sciences, Feinberg School of Medicine, Department of Neurology, Northwestern University, Chicago, IL USA

**Keywords:** Neuropsychological tests, UDS, Alzheimer’s disease, Cognition

## Abstract

**Background:**

Neuropsychological testing plays a cardinal role in the diagnosis and monitoring of Alzheimer’s disease. A major concern is represented by the heterogeneity of the neuropsychological batteries currently adopted in memory clinics and healthcare centers. The current study aimed to solve this issue.

**Methods:**

Following the initiative of the University of Washington’s National Alzheimer’s Coordinating Center (NACC), we presented the Italian adaptation of the Neuropsychological Test Battery of the Uniform Data Set (I-UDSNB). We collected data from 433 healthy Italian individuals and employed regression models to evaluate the impact of demographic variables on the performance, deriving the reference norms.

**Results:**

Higher education and lower age were associated with a better performance in the majority of tests, while sex affected only fluency tests and Digit Span Forward.

**Conclusions:**

The I-UDSNB offers a valuable and harmonized tool for neuropsychological testing in Italy, to be used in clinical and research settings.

**Supplementary Information:**

The online version contains supplementary material available at 10.1186/s13195-022-01056-x.

## Background

Neuropsychological testing plays a central role in the diagnosis of Alzheimer’s disease (AD). The concept of AD as a biological diagnosis based on biomarker positivity has a clear relevance for research, but in most clinical settings, the presence of objective cognitive dysfunction is still representing a “gateway” for a decision about biomarker assessment. The presence of a specific profile of neuropsychological impairment, associated with biomarker positivity, is required for a diagnosis of prodromal AD in a symptomatic individual [14] and is associated with the highest risk of dementia progression [12]. Notwithstanding the key role of neuropsychological assessment for early diagnosis, different tests are employed in memory clinics and healthcare centers, thus introducing heterogeneity in the diagnosis and longitudinal monitoring of AD and mild cognitive impairment (MCI) cases. This aspect constitutes a source of concern when neuropsychological data are shared among different sites, such as in the case of multi-center research projects and consortia.

Attempts to solve this problem through the harmonization of instruments have been pursued, for example, in the USA [25, 26], China [24], and Australia [3]. In Europe, the need for a similar initiative was acknowledged in a consensus conference which recommended as a possible solution the multilingual adaptation of the Neuropsychological Test Battery of the Uniform Data Set (UDSNB) [4]. This battery was designed following the initiative of the University of Washington’s National Alzheimer’s Coordinating Center (NACC), with the initial aim to stage the continuum between normal aging in controls, MCI, and AD patients. The battery underwent revisions and enhancements leading to the currently available paper-and-pencil version 3.0 (UDSNB 3.0), including tests assessing episodic memory, language, executive functions, processing speed, and constructional ability, and has been administered to 3602 healthy controls [25]. Currently, UDSNB has been translated from English and adapted solely for Spanish-speaking individuals [1, 2].

The aim of the present multi-center project is the development of a UDSNB adaptation for the Italian-speaking population (I-UDSNB), including a newly developed tablet-based application to aid the experimenter in test administration and scoring. Here, we report the reference norms obtained from the data collected in a cohort of 433 Italian healthy individuals.

## Methods

### Procedures for battery creation

The initiative, involving 17 centers members of the Virtual Dementia Institute of the RIN (Rete Italiana di Neuroscienze e Neuroriabilitazione-Italian Network of Neuroscience and Neuro-rehabilitation), was supported by a grant from the Italian Ministry of Health. The bases for the creation of the I-UDSNB were discussed during a consensus meeting in February 2020. On that occasion, representatives from six Scientific Institutes for Research, Hospitalization and Healthcare (IRCCS) discussed the feasibility to extend the initiative of the NACC to the Italian context. Permission was requested from the copyright owner, and Prof. Sandra Weintraub accepted to act as an external advisor to the project in the representation of the UDSNB group. The translation/adaptation was based on the American version of UDSNB (UDSNB 3.0, [25]). Then, the six centers participating in the consensus meeting created and revised through feedbacks the materials and the manual specifying the procedures for test administration and scoring. The tablet application was created and underwent a procedure of beta-testing, i.e., the centers tested the application, reporting criticisms and possible improvements to be implemented. A system allowing a web-based data entry was associated to the application, with the possibility to access and download the data via reserved credentials. In February 2021, after the approval of the project by the local ethics committees, the collection of normative data started, involving 14 centers that recruited 433 participants. In November 2021, a working group involving representatives of four centers defined the common procedures and performed the statistical analyses leading to the definition of reference norms.

### Adaptation and development of tests

The I-UDSNB was composed of the following tests (in order of administration, following the American version of UDSNB): Montreal Cognitive Assessment (MoCA), Craft Story, Benson Figure (Copy, Recall), Digit Span Forward and Backward, Semantic Fluency, Trial Making Test A and B (TMT-A, TMT-B), Picture Naming, and Phonemic Fluency. A short encoding controlled, cued recall test (Five Words Test) was added. The administration of the battery takes approximately 45 min. The MoCA data are not reported here, and the examiners may refer to the two available sets of norms to correct the MoCA score for the impact of demographic variables [11, 22]. See Additional file 1 for the description of the tests. The manual, the worksheet, and the tablet application are available upon request to S. Cappa.

### Sample

The recruitment of the normative sample followed the guidelines of Boccardi et al. [4]. Accordingly, age was stratified across five decades (i.e., 40–49, 50–59, 60–69, 70–79, 80–89 years) and education across three levels (i.e., ≤ 8, 9–13, ≥ 14 years), corresponding to compulsory, upper-secondary, and post-secondary educational levels in Italy, respectively. The guidelines recommended to collect data from 10 females and 10 males for each cell in the education/age grid, with the exception of those defined as “rare populations” (i.e., 40–49 years old individuals with ≤ 8 years of education; 80–89 years old individuals with ≥ 14 years of education), the latter allowing to test 5 females and 5 males. We adhered to these recommendations, except for an under-recruitment of older seniors (i.e., 80–89 years). The recruitment was conducted between February and July 2021.

The collected sample included 433 healthy Italian participants (see Table 1). Sex was defined with self-report, see the guidelines of Heidari et al. [18]. Participants were excluded if they had other prior/current neurological or major psychiatric disorders; a history of traumatic brain injury, brain tumors, or stroke; a history of alcohol or drug abuse; a pathological performance in the Mini-Mental State Examination, namely an age- and education-corrected score ≤ 24 (MMSE [19];); sensory or motor deficits possibly affecting performance; and exposure to anesthesia in the previous 3 months.

**Table 1:** Demographics of the normative sample

Number of subjects (females, males)	Age: mean (SD; range)	Education: mean (SD; range)
433 (245, 188)	61.31 (12.79; 40–89)	12.51 (4.49; 2–29)

The number of subjects and the mean, standard deviation, and range of age and education, expressed in years, are reported

*SD* standard deviation

See Table 2 for the demographic data stratified by sex, age, and education. The majority of participants (56.6%) were tested in Northern Italy, followed by Central (30%) and southern-insular regions (13.4%). The study was approved by the local ethics committees and complied with the provisions of the Declaration of Helsinki. All subjects gave written informed consent to participate.

**Table 2:** Distribution of demographic data, stratified by sex, age, and education

Education/age	40–49	50–59	60–69	70–79	80–89	Total F/M	Total
≤ 8	7/6	16/13	17/13	26/11	13/6	79/49	128
9–13	13/17	36/16	22/16	12/12	7/5	90/66	156
≥ 14	25/22	20/14	16/16	14/14	4/4	79/70	149
Total F/M	45/45	72/43	55/45	52/37	24/15		
Total	90	115	100	89	39		433

The number of subjects, as females/males, is reported in each cell

*F* females, *M* males

### Data analysis

We adopted the approach of Capitani and Laiacona [6], commonly used in clinical and research contexts in Italy, to derive the norms. Separately for each test and its sub-scores, simple regression analyses were conducted to evaluate the role of sex, age, and education in predicting the performance. Scores expressed as dichotomous values (e.g., correct/incorrect recognition of Benson figure) were not considered for the analyses described here, but we still explored the effect of demographic variables with binary linear regression models (see Additional file 1).

For age and education, we considered also the logarithmic, i.e., ln(100-age) and ln(30-education); square root; quadratic; and cubic transformations. If different variable transformations resulted significant, we selected the simpler one (e.g., age instead of its transformations) when the difference between the explained variance of the models (expressed as *R*^2^) was smaller than 0.009. Conversely, if the difference between *R*^2^ was greater than 0.009, significant predictors entered in multiple regression and likelihood ratio test were used to select the best fitting model.

Prediction equations were generated from multiple regression analyses including only the predictors significant in the simple regressions. A predictor was included in the final model if significant after multiple regression.

When pertinent, i.e., when the predictor resulted significant in the multiple regression model, the raw score was adjusted to remove the effect of sex, age, and/or education. Minimum and maximum scores were not adjusted, following common procedures adopted for neuropsychological test correction in Italy. Correction grids were generated reversing the signs of beta coefficients derived from the regression model in order to adjust the raw scores by adding/subtracting the effect of the predictors. Adjusted scores were classified into five equivalent scores (ES), from 0 to 4, see Capitani and Laiacona [6] for details. Specifically, cutoffs corresponded to the outer non-parametric tolerance limits with a 95% confidence (corresponding to the 14th observation for 433 participants as in our sample), and values equal or lower/higher than the cutoff value were defined pathological and assigned an ES of 0.

## Results

**Fig. 1:**
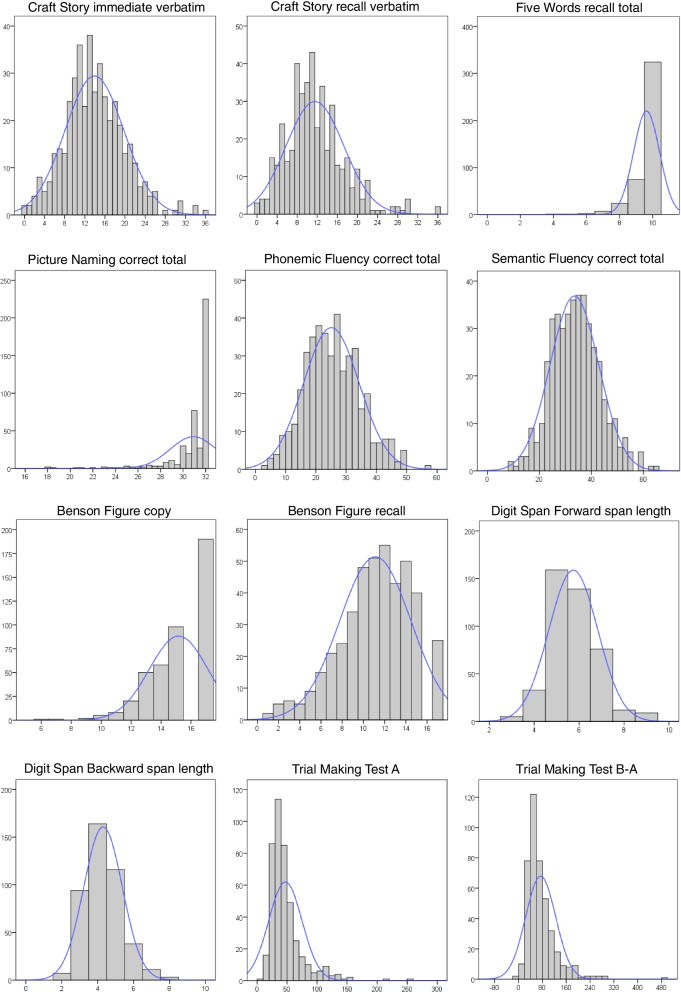
Histograms showing the distributions of the examples of test scores in the battery

Descriptive statistics, cutoff scores for each test, and histograms with examples of score distributions are reported in Table 3 and Fig. 1. See Additional file 1: Table S1 for the correction grids. Data of TMT-B and TMT-B-A were excluded for 4 participants who interrupted the task (part B); the number of correct items in phonemic fluency was not available for one participant due to technical problems in saving the responses.

**Table 3:** Descriptive statistics and cutoff value of each test

Test	Mean (SD)	Min; max	Cutoff (pathological if)	Correction grid
**Craft Story**				
Immediate verbatim score	13.92 (5.88)	0; 36	≤ 4.976	x
Immediate paraphrase score	12.71 (4.08)	2; 22	≤ 6.458	x
Recall verbatim score	11.53 (5.77)	0; 36	≤ 3.128	x
Recall paraphrase score	11.86 (4.24)	0; 22	≤ 5.553	x
**Five Words Test**				
Immediate free recall	4.34 (0.76)	2; 5	≤ 2.831	x
Immediate cued recall	0.59 (0.70)	0; 3	≥ 2.066	x
Immediate total recall	4.93 (0.29)	2; 5	≤ 4	–
Immediate total-weighted	9.27 (0.91)	4; 10	≤ 7.124	x
Delayed free recall	3.92 (1.09)	0; 5	≤ 1.775	x
Delayed cued recall	0.77 (0.84)	0; 4	≥ 2.472	x
Delayed total recall	4.69 (0.64)	1; 5	≤ 3.068	x
Delayed total-weighted	8.61 (1.57)	1; 10	≤ 5.485	x
Total free recall	8.26 (1.54)	2; 10	≤ 5.193	x
Total cued recall	1.36 (1.20)	0; 5	≥ 3.833	x
Total recall	9.63 (0.78)	4; 10	≤ 7.859	x
Total-weighted recall	17.89 (2.13)	7; 20	≤13.335	x
**Picture naming**				
Correct without cue score	30.72 (2.22)	18; 32	≤ 26.241	x
Correct with cue score	0.20 (0.40)	0; 2.5	≥ 1.066	x
Correct total score	30.91 (2.10)	18; 32	≤ 27.329	x
**Semantic fluency**				
Animals’ correct score (< 30 s)	13.47 (4.28)	1; 27	≤ 6.532	x
Animals’ correct score (> 30 s)	7.04 (3.83)	0; 20	≤ 0.164	x
Animals’ total correct score (60 s)	20.51 (6.73)	1; 41	≤ 10.177	x
Animal perseverations	0.70 (1.05)	0; 8	≥ 3	–
Animal violations	0.69 (2.37)	0; 28	≥ 2.846	x
Vegetables’ correct score (< 30 s)	9.36 (3.24)	2; 20	≤ 3.138	x
Vegetables’ correct score (> 30 s)	3.58 (2.35)	0; 12	= 0	x
Vegetables’ total correct score (60 s)	12.94 (4.24)	2; 28	≤ 4.506	x
Vegetable perseverations	0.56 (0.94)	0; 7	≥ 2.760	x
Vegetable violations	0.89 (1.76)	0; 14	≥ 5	–
Total correct score (60 s)	33.44 (9.38)	9; 64	≤ 16.990	x
Total perseverations	1.27 (1.51)	0; 8	≥ 5	–
Total violations	1.58 (3.13)	0; 28	≥ 10.715	x
**Phonemic fluency**				
Letter F correct score (< 30 s)	8.81 (3.42)	2; 24	≤ 3.746	x
Letter F correct score (> 30 s)	5.09 (2.72)	0; 14	≤ 0.718	x
Letter F total correct score (60 s)	13.91 (5.14)	2; 31	≤ 6.747	x
Letter F perseverations	0.47 (0.84)	0; 8	≥ 2	–
Letter F violations	0.33 (0.81)	0; 6	≥ 3	–
Letter L correct score (< 30 s)	7.29 (3.14)	0; 21	≤ 2.346	x
Letter L correct score (> 30 s)	3.91 (2.48)	0; 11	= 0	x
Letter L total correct score (60 s)	11.20 (4.79)	0; 27	≤ 3.520	x
Letter L perseverations	0.48 (0.91)	0; 8	≥ 2	–
Letter L violations	0.41 (0.85)	0; 7	≥ 3	–
Total correct score (60 s)	25.11 (9.22)	3; 56	≤ 10.888	x
Total perseverations	0.94 (1.46)	0; 14	≥ 3.980	x
Total violations	0.74 (1.46)	0; 13	–	–
**Benson figure**				
Copy	15.18 (1.96)	6; 17	≤ 11.931	x
Recall	11.12 (3.36)	1; 17	≤ 5.481	x
**Digit Span Forward**				
Number of correct trials	6.41 (1.98)	1; 13	≤ 3.519	x
Span length	5.74 (1.09)	3; 9	≤ 3.917	x
**Digit Span Backward**				
Number of correct trials	5.78 (1.86)	1; 13	≤ 3.023	x
Span length	4.30 (1.07)	1; 8	≤ 2.751	x
**Trial Making Test**				
Part A (s)	47.11 (27.89)	7; 252	≥ 93.699	x
Part B (s)	121.08 (67.94)	21; 613	≥ 226.340	x
Parts B-A (s)	74.08 (50.46)	-19; 481	≥ 177.600	x

Mean (and standard deviation), minimum and maximum scores, and cutoff value for each test of the battery

*min* minimum score, *max* maximum score, *x* correction grid available, “–” no correction grid available

### Craft Story

Age influenced negatively the immediate verbatim score (*β* = − 0.065, SE = 0.021). Education influenced positively the immediate verbatim score (*β* = 0.500, SE = 0.059). Immediate paraphrase was predicted by the quadratic function of education (education: *β* = 0.836, SE = 0.168; education^2^: *β* = − 0.018, SE = 0.007). Age negatively predicted the performance in recall verbatim (*β* = − 0.104, SE = 0.021) and recall paraphrase (*β* = − 0.042, SE = 0.0149). Education positively predicted the performance in recall verbatim (*β* = 0.391, SE = 0.058) and recall paraphrase (*β* = 0.378, SE = 0.043).

### Five Words Test

#### Immediate recall

Age negatively predicted the immediate free recall (*β* = − 0.0127, SE = 0.003). The immediate cued recall was positively predicted by age (*β* = 0.013, SE = 0.003). Notably, in cued recall, the highest scores reflect the worst performance, thus suggesting a difficulty in spontaneously recalling the items resulting in the need for the cue, i.e., the semantic category to which the item belongs, for the correct retrieval. No demographic variables influenced immediate total recall, while immediate total-weighted recall was negatively predicted by age (*β* = − 0.013, SE = 0.004).

#### Delayed recall

Delayed free recall was negatively predicted by age (*β* = − 0.025, SE = 0.004) and by the cubic function of education (education: *β* = 0.427, SE = 0.128; education^2^: *β* = − 0.026, SE = 0.010; education^3^: *β* = 0.0001, SE = 0.0002). Age positively predicted delayed cued recall (*β* = 0.015, SE = 0.003). Education negatively predicted delayed cued recall (*β* = − 0.0311, SE = 0.009). Delayed total recall and delayed total-weighted recall were negatively predicted by age (*β* = − 0.011, SE = 0.002; *β* = − 0.036, SE = 0.006, respectively) and by the cubic function of education (education: *β* = 0.239, SE = 0.079, education^2^: *β* = − 0.015, SE = 0.006, education^3^: *β* = 0.0003, SE = 0.0001; education: *β* = 0.666, SE = 0.185, education^2^: *β* = − 0.041, SE = 0.014, education^3^: *β* = 0.001, SE = 0.0003, respectively).

#### Total recall

Total free recall was negatively predicted by age (*β* = − 0.037, SE = 0.006) and by the cubic function of education (education: *β* = 0.528, SE = 0.181, education^2^: *β* = − 0.031, SE = 0.0134, education^3^: *β* = 0.0006, SE = 0.0003). Total cued recalled was positively predicted by age (*β* = 0.027, SE = 0.004) and negatively predicted by education (*β* = − 0.045, SE = 0.0123). Total recall was negatively predicted by age (*β* = − 0.012, SE = 0.003) and by the quadratic function of education (education: *β* = 0.084, SE = 0.036, education^2^: *β* = − 0.003, SE = 0.001). Age negatively predicted delayed total-weighted recall (*β* = − 0.048, SE = 0.008).

### Picture naming

The quadratic function of age (age: *β* = 0.200, SE = 0.068, age^2^: *β* = − 0.002, SE = 0.0005) and the cubic function of education (education: *β* = 1.141, SE = 0.248, education^2^: *β* = − 0.061, SE = 0.019, education^3^: *β* = 0.001, SE = 0.0004) predicted the correct without cue score. The correct with cue score was predicted positively by age (*β* = 0.006, SE = 0.002) and negatively by education (*β* = − 0.015, SE = 0.004). An increase in the latter score indicated a worse performance, since it considers the number of cues given during the task when participants were unable to spontaneously name the pictures. The correct total score was predicted by the quadratic function of age (age: *β* = 0.182, SE = 0.063, age^2^: *β* = − 0.002, SE = 0.0005) and the cubic function of education (education: *β* = 1.095, SE = 0.231, education^2^: *β* = − 0.059, SE = 0.017, education^3^: *β* = 0.001, SE = 0.0004).

### Fluency

#### Semantic fluency

The correct score for the animal category (< 30 s) was predicted negatively by age (*β* = − 0.084, SE = 0.015) and was predicted positively by education (*β* = 0.254, SE = 0.044). Animals’ correct score (> 30 s) and animals’ total correct score (60 s) were negatively predicted by age (*β* = − 0.030, SE = 0.014; *β* = − 0.112, SE = 0.024, respectively) and by the quadratic function of education (education: *β* = 0.591, SE = 0.169, education^2^: *β* = − 0.014, SE = 0.007; education: *β* = 1.167, SE = 0.281, education^2^: *β* = − 0.027, SE = 0.011, respectively). Violations were negatively predicted by age (*β* = − 0.035, SE = 0.009). No demographic variables predicted the number of perseverations.

Sex (*β* = 1.931, SE = 0.290), age (*β* = − 0.050, SE = 0.012), and education (*β* = 0.236, SE = 0.033) impacted the vegetables’ category correct score (< 30 s): females performed better than males; performance was negatively predicted by age and positively predicted by education. Vegetables’ correct score (> 30 s) was positively predicted by education (*β* = 0.058, SE = 0.025) and by sex (*β* = 0.577, SE = 0.227), with females performing better than males. Vegetables’ total correct score (60 s) was predicted by sex (*β* = 2.514, SE = 0.381), age (*β* = − 0.055, SE = 0.015), and education (*β* = 0.189, SE = 0.044), similarly to vegetables’ correct score (< 30 s). Females produced more perseverations than males (*β* = 0.238, SE = 0.091), while no variables influenced the number of violations.

Semantic fluency total correct score (60 s) was negatively predicted by age (*β* = − 0.167, SE = 0.033), quadratic function of education (education: *β* = 1.476, SE = 0.387, education^2^: *β* = − 0.032, SE = 0.015), and sex, with females performing better than males (*β* = 3.071, SE = 0.808). The total number of violations was negatively predicted by age (*β* = − 0.034, SE = 0.012), while no demographic variables influenced the total number of perseverations.

#### Phonemic fluency

Letter F correct score (< 30 s) was negatively predicted by age (*β* = − 0.064, SE = 0.011), the quadratic function of education (education: *β* = 0.574, SE = 0.135, education^2^: *β* = − 0.011, SE = 0.005), and sex, with females performing better than males (*β* = 1.034, SE = 0.282). Letter F correct score (> 30 s) and letter F total correct score (60 s) were negatively predicted by age (*β* = − 0.027, SE = 0.010; *β* = − 0.091, SE = 0.017, respectively) and positively predicted by education (*β* = 0.224, SE = 0.028; *β* = 0.517, SE = 0.048, respectively). No demographic variables influenced the number of perseverations and violations in letter F fluency.

Letter L correct score (< 30 s) was predicted negatively by age (*β* = − 0.043, SE = 0.011) and positively by education (*β* = 0.285, SE = 0.031) and sex, with females performing better than males (*β* = 1.058, SE = 0.266). Letter L correct score (> 30 s) was predicted negatively by age (*β* = − 0.025, SE = 0.009) and positively by education (*β* = 0.197, SE = 0.025). Age (*β* = − 0.068, SE = 0.016), education (*β* = 0.484, SE = 0.045), and sex (*β* = 1.427, SE = 0.394) predicted letter L total correct score (60s), similarly to letter L correct score (< 30 s). No demographic variables influenced the number of perseverations and violations in letter L fluency.

Phonemic fluency total correct score (60 s) was influenced negatively by age (*β* = − 0.151, SE = 0.030) and positively by the square root of education (*β* = 6.686, SE = 0.559). Education positively influenced the total number of perseverations in phonemic fluency (*β* = 0.041, SE = 0.016), while no variable influenced the total number of violations.

### Benson figure

Performance in the copy of Benson figure was predicted by the cubic function of education (education: *β* = 1.124, SE = 0.230, education^2^: *β* = − 0.066, SE = 0.017, education^3^: *β* = 0.001, SE = 0.0004), while performance in the recall was negatively predicted by age (*β* = − 0.084, SE = 0.012) and by the quadratic function of education (education: *β* = 0.591, SE = 0.138, education^2^: *β* = − 0.017, SE = 0.005).

### Digit Span Forward

Considering the number of correct trials, females performed worse than males (*β* = − 0.346, SE = 0.172), and performance was negatively predicted by age (*β* = − 0.027, SE = 0.007) and by the quadratic function of education (education: *β* = 0.324, SE = 0.082, education^2^: *β* = − 0.007, SE = 0.003). Span length was predicted by the cubic function of age (age: *β* = − 0.605, SE = 0.251, age^2^: *β* = 0.009, SE = 0.004, age^3^: *β* = 0.00005, SE = 0.00002) and by the quadratic function of education (education: *β* = 0.165, SE = 0.047, education^2^: *β* = − 0.004, SE = 0.002).

### Digit Span Backward

The number of correct trials was predicted by the quadratic function of age (age: *β* = − 0.172, SE = 0.058, age^2^: *β* = 0.001, SE = 0.0004) and education (education: *β* = 0.331, SE = 0.077; education^2^: *β* = − 0.008, SE = 0.003), while span length was predicted by the quadratic function of age (age: *β* = − 0.087, SE = 0.034, age^2^: *β* = 0.0005, SE = 0.0003) and by the square root function of education (*β* = 0.494, SE = 0.072).

### TMT

TMT-A was predicted by the cubic function of age (age: *β* = 13.089, SE = 5.446, age^2^: *β* = − 0.220, SE = 0.088, age^3^: *β* = 0.001, SE = 0.0005) and education (education: *β* = − 14.095, SE = 2.848, education^2^: *β* = 0.745, SE = 0.213, education^3^: *β* = − 0.012, SE = 0.005). TMT-B was predicted by the quadratic function of age (age: *β* = − 4.758, SE = 1.795, age^2^: *β* = 0.055, SE = 0.0143) and the cubic function of education (education: *β* = − 33.907, SE = 6.816, education^2^: *β* = 1.737, SE = 0.507, education^3^: *β* = − 0.029, SE = 0.012). TMT-B-A was negatively predicted by the logarithmic function of age (*β* = − 46.805, SE = 5.412) and by the quadratic function of education (education: *β* = − 10.921, SE = 1.980, education^2^: *β* = 0.293, SE = 0.077).

## Discussion

The current study reports the Italian adaptation of the Uniform Data Set Neuropsychological Test Battery, offering normative data from a cohort of 433 healthy Italian individuals. It represents the second adaptation of the UDSNB for non-English speaking individuals, following the analogous initiative for Spanish-speaking ones [1, 2].

The Italian-speaking cohort included more participants (*n* = 433) than the Spanish one (*n* = 276) [2] but fewer than the English-speaking cohorts (*n* = 3602) [25]. Our participants were younger than both Spanish and English ones, i.e., 61.31 years old compared to 70 and 74, respectively, with an education in-between the two, i.e., 12.51 years compared to 10.7 and 15.7. All cohorts have a higher proportion of females than of males, but with different percentages, i.e., 56%, 63%, and 76% in Italian, English, and Spanish speaking cohorts, respectively.

Differently from the currently available versions of the battery, in the I-UDSNB we have introduced the use of the tablet, as a tool to help the examiner in administering the tests, in recording the responses, and in attributing the scores. Notably, the participants did not have a direct interaction with the tablet, so we did not expect any major effect of its introduction in the overall performance of our sample. As consequence, the slight differences between the current Italian version and the two previous ones in terms of the effect of demographic variables on the performance (detailed below) were unlikely to be attributable to the introduction of the tablet.

The majority of tests in the I-UDSNB were translated/adapted from the US version. The same scoring procedures were adopted as well, with minimal differences. Following the procedures employed in Italy [15], in TMT, we did not include the indication of the correct lines/time; moreover, in fluency tests, we also separately counted the number of items produced in the first and in the last 30 s.

In line with the US version, the score distributions of correct responses in the Picture Naming Test, Benson Figure Copy, and Five Words Test were skewed due to the presence of ceiling effects, likely resulting from an overrepresentation of young and highly educated individuals.

In good agreement with the other cohorts [2, 25], demographic factors (age, sex, education) affected some of the I-UDSNB sub-scores. Younger individuals and those with higher education performed better in the I-UDSNB tests assessing episodic memory (Craft Story, Benson Figure Recall), language functions (picture naming, fluency), visuo-constructional abilities (Benson Figure Copy), short-term memory (Digit Span Forward), and attention and executive functions (TMT, Digit Span Backward). These effects were in line with other studies testing Italian samples [5, 9, 10, 15, 20, 21, 23]. The number of violations in semantic fluency was negatively predicted by age, while the perseverations in phonemic fluency were positively predicted by education. Notably, age did not influence the immediate paraphrase recall of the Craft Story, the copy of the Benson figure, and the number of correct vegetables (> 30 s).

These effects are in line with those obtained with American UDSNB, except for an additional impact of age on the immediate paraphrase recall of the Craft Story and on the copy of the Benson figure. It is noteworthy that, in the Spanish UDSNB, the effect of age was instead restricted to the delayed recall of Craft Story, TMT-B, and category fluency (vegetables); as suggested by the authors, this finding might result from a smaller sample compared to the original study on English-speaking participants.

Considering the newly added Five Words Test, an effect of age was reported for immediate recall, while both age and education influenced delayed and total recall. In the cued condition, the score increased with age and decreased with education, because older and less educated individuals tended to spontaneously recall fewer items in the free recall condition, thus needing the semantic cue for the correct retrieval. Notably, contrary to other Italian versions of the Free and Cued Selective Reminding Test [13, 16], in our study, the impact of demographic variables was not circumscribed to free, but extended to cued and total, recall. Such inconsistencies may depend on the differences in both the stimuli used (i.e., 5 words in I-UDSNB, 12 pictures in Frasson et al. [13] and 16 words in Girtler et al. [16]) and sample size (i.e., 433 (I-UDSNB) vs. 194 [16] or 227 [13] participants).

Considering the sex effects, in line with the US version of the UDSNB and with previous studies in Italian cohorts [7, 8, 17], we found that males performed better in the Digit Span Forward, while females outperformed males in phonemic and category fluency. In the latter, females showed also more perseverations than males. These findings highlight controversial evidence regarding the sex effect in the available literature. Indeed, differently from our results, in the US version of the UDSNB females outperformed males also in the Craft Story, while performing worse in the recall of the Benson figure. In the naming test, females performed worse in the US but better in the Spanish UDSNB. In agreement with our results, no sex effects were found in verbal episodic memory [23] and naming tests [9]. Mixed evidence emerged, instead, in the recall of the Benson figure, with a previous Italian study reporting an advantage for females [5]. Heterogeneity may be ascribed to the differences in stimuli complexity (i.e., Benson vs. Rey-Osterrieth figure) and sample size (i.e., 433 vs. 280 individuals).

## Conclusions

Since 2005, the NACC has collected the Uniform Data Set on participants from over 30 US Alzheimer’s Disease Centers. The dataset includes a wealth of data, which are available for sharing and provide a rich source for hypothesis generation and investigation in cognitive aging and dementia. A central component of this project is represented by the uniform neuropsychological test battery (UDSNB), whose most recent revision (UDSNB 3.0) was published in 2018. In an effort to harmonize neuropsychological assessment in Europe, the UDSNB 3.0 was considered an excellent model for the development of a test battery for AD diagnosis in memory clinics [4]. This initiative inspired the present work, aiming at the development of a test battery to be used as part of the common dataset of the Virtual Dementia Institute of the Italian Neuroscience and Rehabilitation Network founded in 2017 by the Italian Ministry of Health. The I-UDSNB includes tests assessing the cognitive domains that are known to be compromised in AD from the early (prodromal) stages, capturing the early symptoms of cognitive decline in older individuals. The availability of norms allows to use the I-UDSNB in clinical and research settings, while controlling for the impact of age, education, and sex on performance. As the main advantage of this work, the battery stands as a useful harmonized neuropsychological tool that can be adopted in multicenter studies for the initial assessment and monitoring of MCI and AD patients.

One limitation of the current study is the distribution of our sample. In the first place, the older participants were under-represented, in line with previous normative studies reporting difficulty in sampling this population and as recently also stressed by Boccardi et al. [4]. Second, we did not manage to collect participants from all the Italian regions, as a consequence of the geographical distribution of the centers involved in the project as part of the Italian Neuroscience and Rehabilitation Network. Our sample, however, included individuals from the three macro-areas of Italy, namely northern, central, and southern-insular regions.

Future steps will proceed in three directions. First, the validity of the battery will be formally tested in MCI and AD individuals. Second, the potentiality of tablet-based application will be expanded by the development of a fully computerized battery for remote administration. Third, the aims of the NACC initiative will be pursued further via the design and development of parallel modules for the diagnosis of other forms of dementia such as fronto-temporal lobar degeneration and Lewy bodies dementia.

## Supplementary Information

**Additional file 1: **Supplementary materials. **Table S1.** Correction grids for age, education and sex and Equivalent Scores for each test (when available). To correct the raw score, the examiner has to add/subtract the values indicated on the bases of the subject age and/or education and/or sex to the raw score. Corrected score is then assigned an Equivalent Score (when available) according to the corresponding grid of values.

## Data Availability

The datasets used and/or analyzed during the current study are available from the corresponding author on reasonable request.

## Abbreviations

NACC: University of Washington’s National Alzheimer’s Coordinating Center
I-UDSNB: Italian adaptation of Neuropsychological Test Battery of the Uniform Data Set
AD: Alzheimer’s disease
MCI: Mild cognitive impairment
UDSNB: Neuropsychological Test Battery of the Uniform Data Set
IRCCS: Scientific Institutes for Research, Hospitalization and Healthcare
MoCA: Montreal Cognitive Assessment
TMT: Trial Making Test
MMSE: Mini-Mental State Examination
SD: Standard deviation
ES: Equivalent scores
