# Correction: Italian adaptation of the Uniform Data Set Neuropsychological Test Battery (I‑UDSNB 1.0): development and normative data

**DOI:** 10.1186/s13195-023-01247-0

**Published:** 2023-06-05

**Authors:** Francesca Conca, Valentina Esposito, Francesco Rundo, Davide Quaranta, Cristina Muscio, Rosa Manenti, Giulia Caruso, Ugo Lucca, Alessia Antonella Galbussera, Sonia Di Tella, Francesca Baglio, Federica L’Abbate, Elisa Canu, Valentina Catania, Massimo Filippi, Giulia Mattavelli, Barbara Poletti, Vincenzo Silani, Raffaele Lodi, Maddalena De Matteis, Michelangelo Stanzani Maserati, Andrea Arighi, Emanuela Rotondo, Antonio Tanzilli, Andrea Pace, Federica Garramone, Carlo Cavaliere, Matteo Pardini, Cristiano Rizzetto, Sandro Sorbi, Roberta Perri, Pietro Tiraboschi, Nicola Canessa, Maria Cotelli, Raffaele Ferri, Sandra Weintraub, Camillo Marra, Fabrizio Tagliavini, Eleonora Catricalà, Stefano Francesco Cappa

**Affiliations:** 1 IRCCS Mondino Foundation, Pavia, Italy; 2 Department of Neurology IC, Oasi Research Institute – IRCCS, Troina, Italy; 3 Neurology Unit, Fondazione Policlinico Universitario “A. Gemelli” IRCCS, Rome, Italy; 4 Department of Psychology, Catholic University of the Sacred Heart, Milan, Italy; 5 Present address: ASST Bergamo Ovest, Treviglio, Italy; 6 Fondazione IRCCS Istituto Neurologico Carlo Besta, Milan, Italy; 7 IRCCS Istituto Centro San Giovanni Di Dio Fatebenefratelli, Brescia, Italy; 8 Laboratory of Clinical and Behavioural Neurology, IRCCS Santa Lucia Foundation, Rome, Italy; 9 Laboratory of Geriatric Neuropsychiatry, Department of Neuroscience, Istituto Di Ricerche Farmacologiche Mario Negri IRCCS, Milan, Italy; 10 IRCCS Fondazione Don Carlo Gnocchi, ONLUS, Milan, Italy; 11 Neuroimaging Research Unit, Division of Neuroscience, IRCCS San Raffaele Scientific Institute, Milan, Italy; 12 Unit of Psychology I.C., Oasi Research Institute–IRCCS, Troina, Italy; 13 Neurology Unit, Neurorehabilitation Unit, and Neurophysiology Service, IRCCS San Raffaele Scientific Institute, Vita-Salute San Raffaele University, Milan, Italy; 14 IUSS Cognitive Neuroscience (ICON) Center, Scuola Universitaria Superiore IUSS, Palazzo del Broletto, Piazza Vittoria 15, 27100 Pavia, Italy; 15 Istituti Clinici Scientifici Maugeri IRCCS, Cognitive Neuroscience Laboratory of Pavia Institute, Pavia, Italy; 16 Department of Neurology and Laboratory of Neuroscience, IRCCS Istituto Auxologico Italiano, Milan, Italy; 17 Aldo Ravelli Research Center for Neurotechnology and Experimental Brain Therapeutics, Università Degli Studi Di Milano, Milan, Italy; 18 IRCCS Istituto Delle Scienze Neurologiche Di Bologna, Bologna, Italy; 19 Fondazione IRCSS Ca’ Granda, Ospedale Policlinico, Milan, Italy; 20 Neuro-Oncology Unit, IRCCS Regina Elena National Cancer Institute, Rome, Italy; 21 IRCCS Synlab SDN of Naples, Naples, Italy; 22 IRCCS Ospedale Policlinico San Martino, Genoa, Italy; 23 Department of Neuroscience (DINOGMI), University of Genoa, Genoa, Italy; 24 Mesulam Center for Cognitive Neurology and Alzheimer’s Disease and Department of Psychiatry and Behavioral Sciences, Feinberg School of Medicine, Department of Neurology, Northwestern University, Chicago, IL USA

**Correction: Alz Res Therapy 14, 113 (2022)**

**https://doi.org/10.1186/s13195-022-01056-x**

Following publication of the original article [1], the authors deleted affiliation 18 of the author Vincenzo Silani^16,17,18^ for the reason that the funding agency (Italian Ministry of Health) accepts a maximum of 2 affiliations for each researcher.

The original article [1] has been updated.
