# Supplementary material for: Italian adaptation of the Uniform Data Set Neuropsychological Test Battery (I-UDSNB 1.0): development and normative data
Source: Alzheimers Res Ther. 2022 Aug 19;14:113. doi: 10.1186/s13195-022-01056-x (PMC9389755; doi:10.1186/s13195-022-01056-x)
Supplement: Supplementary file 1 — Additional file 1: Supplementary materials. Table S1. Correction grids for age, education and sex and Equivalent Scores for each test (when available). To correct the raw score, the examiner has to add/subtract the values indicated on the bases of the subject age and/or education and/or sex to the raw score. Corrected score is then assigned an Equivalent Score (when available) according to the corresponding grid of values. [file 13195_2022_1056_MOESM1_ESM.docx]

**Supplementary materials**

**Adaptation and development of neuropsychological tests**

***Episodic Memory***

*Craft story.* The Craft Story was adapted from the American version, privileging a simple and colloquial structure rather than a literal translation. The two proper names (“Maria” and “Ricky”) of the American story were replaced with other two proper names of comparable frequency (i.e. respectively assigned to the 0.08% and 0.01% of the newborns in USA and Italy) and length (i.e. 5 letters) in Italian, i.e. “Carla” and “Ennio”. The participant was read the story and asked to recall it immediately (immediate recall) and 20 minutes later (delayed recall). If the participant failed to spontaneously recall the story, a phonemic cue was given. The Verbatim and Paraphrase scores were recorded. For the Verbatim score, one point was given for each word that had been recalled exactly (maximum of 36), while for the Paraphrase score one point was given for a response that captured the meaning of the original words, although not being strictly identical (maximum of 22).

*Five Words Test*. The Five Words Test is the only addition to the original UDSNB 3.0. This choice was motivated by the interest in a more extensive assessment of verbal episodic memory. Indeed, convergent literature stressed the capability of tests assessing verbal episodic memory to show a high predictive accuracy in tracking the progression from MCI to AD (Belleville et al., 2017).

Different versions of the test existed, that we used here to guide the creation of the current one, including two in French (Dubois et al., 2002, see Rozzini et al., 2017 for the Italian translation; Croisile et al., 2007) and two Italian versions of the free and cued selective reminding test (FCSRT, Girtler et al., 2015; Frasson et al., 2011). Those tests differed for the number of items, namely 5 (Dubois et al., 2002; Croisile et al., 2007) or 16 (Girtler et al., 2015; Frasson et al., 2011), the type of stimuli, namely words (Dubois et al., 2002; Croisile et al., 2007; Girtler et al., 2015) or pictures (Frasson et al., 2011), and for the semantic categories adopted, with some categories present in three versions (e.g. flower, fruit, musical instrument in Croisile et al., 2007; Girtler et al., 2015; Frasson et al., 2011), while other present only in one (e.g. insect in Dubois et al., 2002; sport in Girtler et al., 2015). Here, we decided to use five printed words, namely stork (*cicogna*), asparagus (*asparago*), mimosa (*mimosa*), accordion (*fisarmonica*), tractor (*trattore*) (number of letters: mean 8, range 6-11; number of syllables: mean 3.60, range 3-5; number of phonemes: mean 8.67, range 7-11; values for PhonItalia, Goslin et al., 2014). They indicated items belonging to the categories of, respectively, birds, vegetables, flowers, musical instruments and means of transport. Typicality and word frequency were used for items’ selection. The items were chosen to have a low typicality (mean 3.20, range: 2.8-3.6, on a scale from 1= not typical, to 7= highly typical; Dell’Acqua et al., 2000), to prevent, during cued recall, a randomly responding participant to give a correct response only because the requested item was prototypical for the category, e.g. as a dog for the category of animals. Typicality was slightly higher in the two Italian FCSRT, with a mean of 5.04 (range 4.1-5.9) in Girtler et al. (2015) and 5.65 (range 2.7-6.9) in Frasson et al. (2011) (Dell’Acqua et al., 2000). In the two French versions, typicality was derived from an unpublished work by Dubois (1986) and we couldn’t make an exact comparison with the Italian values in Dell’Acqua et al. (2000). Notably, mean typicality was respectively of 21.1 (Dubois et al., 2002) and 35 (Croisile et al., 2007), and authors specified to have selected not typical items, by excluding the 5 or 6 most typical exemplars for each category. Taking into account word frequency, we excluded infrequent items, namely belonging to the 1^st^ quartile of frequency distribution in the database of Goslin et al. (2014), and the items we used had a mean frequency of 1.68 (range 0.69-2.56, log absolute frequency). Also the items in the French versions did not belong to the 1^st^ quartile of frequency distribution in the Brulex database (Content et al., 1990) (mean= 1098, range= 51-2761 in Dubois et al., 2002; mean= 452, range= 153-791 in Croisile et al., 2007). As for the Italian tests, all items in Frasson et al. (2011) belonged to the 4^th^ quartile of frequency, i.e. they were the most frequent ones (mean= 4, range= 2.83-6.40), while the items of Girtler et al. (2015) were distributed in all the four quartiles (mean= 2.13, range= 0-3.87) (Goslin et al., 2014).

The procedures and instructions for test administration were adapted from Dubois et al.’s (2002) “Le 5 mots test”, and consisted of an initial encoding phase of 5 written words, followed by the immediate and delayed (5 minutes later) recall. In both recall phases, if the participant failed to freely recall an item, the corresponding cue was given, i.e. the semantic category of the item. Separately for immediate, delayed and total (i.e. sum of immediate and delayed) recall, we recorded: a) free recall, b) cued recall, c) total recall, i.e. sum of free and cued recall, d) total-weighted recall, i.e. sum of (2 x free recall) and cued recall.

*Benson Figure Recall*. The recall of the Benson Figure (Possin et al., 2011) was used to assess non-verbal episodic memory. After 15 minutes from the copy, the participants were asked to reproduce the figure in a maximum of 4 minutes. The scoring was identical to the Benson Figure Copy described below.

***Language***

*Picture naming*. Picture naming was created referring to the 32-items Multi-Lingual Naming Test (MINT-32) (Gollan et al., 2012; Ivanova et al., 2013) used in the American UDSNB. In addition to the version in English, the MINT exists also in Spanish, Hebrew and Mandarin Chinese, and was designed to assess the naming abilities in bilinguals, taking into account both dominant and non-dominant language performance, guaranteeing an equivalent difficulty of items across different languages. In the American UDSNB, the items of the MINT-32 were selected from a longer version (68-items), and consisted of items of medium difficulty. The latter criterion, i.e. selection of items with medium, rather than high difficulty, might be important to assess naming abilities in a non-dominant language but it might be less specific in distinguishing between patients and healthy controls. Of note, MINT-32, although showing sensitivity to naming impairment in AD, did not provide acceptable diagnostic accuracy in discriminating between MCI and healthy controls (Stasenko et al., 2019). Accordingly, stimuli for the current Italian version were selected across all the frequency distribution, namely including items with low (1^st^ quartile), medium (2^nd^ and 3^rd^ quartile) and high (4^th^ quartile) frequency. Selected stimuli were 32 black-and-white line drawings (International Naming Project; Bates et al., 2000), of which 10 with high frequency (frequency > 1.94, values of word frequency taken from CoLFIS), 13 with medium frequency (frequency between 0.69 and 1.94) and 9 with low frequency (frequency equal to 0). Stimuli had a mean visual complexity of 17802.13 (range= 4325-62223, information obtained from the size of the digitalized picture; Bates et al., 2000), name agreement of 0.51 (range= 0-1.81, with 0 indicating a perfect name agreement; Bates et al., 2000), typicality of 4.29 (range= 1.20-6.30, on a scale from 1= not typical, to 7= highly typical; Dell’ Acqua et al., 2000), Age of Acquisition of 3.75 (range= 2.40-6.10, on a scale from 1= acquired at 2 years old or younger, to 9= acquired at 13 years old or older; Dell’ Acqua et al., 2000), familiarity of 4.47 (range= 2.70-6.10, on a scale from 1= not familiar, to 7= highly familiar; Dell’Acqua et al., 2000), number of letters of 7.31 (range= 4-10) and syllables of 3 (range= 2-5; database PhonItalia, Goslin et al., 2014), imageability of 6.11 (range= 5.84-6.43, on a scale from 1= not imageable, to 7= highly imageable; Barca et al., 2002) and mean number of meaning of 1.56 (range= 1-6; WordNet database).

Each picture was presented for 6 s, and a phonological cue was given if the participant was unable to name it within this time limit. The test stopped after 6 consecutive failures. Three scores were recorded: a) correct without cue, i.e. 1 point for each correct response; b) correct with cue, i.e. 0.5 points for each correct response after the cue; c) correct total, i.e. sum of correct responses without and with cue (range 0-32).

*Fluency (semantic and phonemic)*. Semantic and phonemic fluency, translated from to the US version, were used as a measure of word retrieval, while additionally tracking performance in semantic memory and executive functions, respectively. In semantic fluency test, participants were asked to produce as many different exemplars of the two semantic categories (animals and vegetables) as possible, each within 1 minute. In the phonemic fluency test, they were asked to produce as many different words beginning with the letter “F” and the letter “L” as possible, each within 1 minute. The two semantic categories, i.e. animals and vegetables, and the two letters, i.e. F and L, were also used in the US version and present in fluency tests in Italian clinical and research practice (Novelli et al., 1986). The correct score included only the unique exemplars produced, while perseverations (i.e. repetitions) and violations (i.e. exemplars not belonging to animals or vegetables categories) were recorded apart. For both semantic (i.e. separately for animals and vegetables) and phonemic (i.e. separately for F and L letters) fluency tests, we also recorded the number of words generated in the first 30 s and in the second 30 s.

***Visuo-constructional abilities***

*Benson Figure Copy.* The copy of the Benson Figure was taken from Possin et al. (2011) and adopted to assess visuo-constructional abilities. Participants were presented the figure and had to copy it in a maximum of 4 minutes. The 16 elements of the figure were scored for presence/absence and for correct/incorrect placement. A 1-point bonus was given if all the elements were present and correctly placed.

***Short term memory***

*Digit Span Forward.* Digit Span Forward, translated from the US version, was used to assess verbal short term memory. Participants had to repeat the number list in the same order they were presented, and the task continued up to the failure on two trials with the same length. The total number of correct trials and the span-length (i.e., the longest repeated list), were recorded.

***Attention and executive functions***

*Digit Span Backward*. This test, translated from the US version, was used to evaluate working memory. Participants had to repeat the number list while reversing the order with which they were presented. The scoring was identical to the Digit Span Forward.

*Trial Making Test.* Trial Making Test (TMT) was taken from the available version of Giovagnoli et al. (1996) and used to evaluate visual attention, processing speed and executive functions. The test consisted of part A and part B. In the TMT-A, participants were asked to connect with a line 25 stimuli, i.e. numbers, in ascending order and in the shortest possible time. In the TMT-B, they had to connect with a line 25 stimuli, i.e. numbers and letters, in ascending and alternating order and in the shortest possible time. The time (in seconds) needed to complete part A, part B and the difference between the two, was recorded.

**Tests administration**

The tests of the I-UDSNB were administered according to three different modalities consistently among participants, i.e. all the participants completed the battery using the same administration modality:

1) Only paper and pencil: MoCA;

2) Only tablet: Digit Span Forward and Backward; Fluency tests;

3) Both using paper and pencil and tablet: Craft Story; Benson Figure; Five Words Test; Trial Making Test; Naming Test. This latter modality means that the tablet was used to help the examiner in administering the test. For example, in the case of the Naming Test, the pictures were presented in a printed sheet of paper, while the examiner took note of the time, the responses and eventual use of the cue using the tablet.

See manual and worksheet (available upon request) for additional information.

**Effect of demographic variables on dichotomous scores**

In the I-UDSNB two scores were expressed as dichotomous values, namely the recognition of the Benson figure (i.e. 0= incorrect recognition, 1= correct recognition) and the use of the cue in the Craft Story (i.e. 0= cue not required, 1= cue required). These scores were not adjusted for the effect of demographic variables (see main text), but we still explored the impact of age, education and sex on the scores, by means of binary logistic regressions models. We found an effect of age (unstandardized B: -0.027, SE: 0.013) and education (unstandardized B: 0.086, SE: 0.037) on the recognition of the Benson Figure. Specifically, younger and highly educated participants had a better recognition than older and less educated ones. We also found an effect of age (unstandardized B: 0.103, SE: 0.030) and education (unstandardized B: -0.152, SE: 0.068) on the use of the cue in the Craft Story. Indeed, older and less educated participants required the cue more frequently than younger and higher educated ones.

**Supplementary References**

Barca, L., Burani, C., & Arduino, L. S. (2002). Word naming times and psycholinguistic norms for Italian nouns. *Behavior research methods, instruments, & computers*, *34*(3), 424-434.

Bates, E., Andonova, E., D’Amico, S., Jacobsen, T., Kohnert, K., Lu, C., ... & Pléh, C. (2000). Introducing the CRL international picture-naming project (CRL-IPNP). Center for Research in Language Newsletter, 12(1), 1-14.

Belleville, S., Fouquet, C., Hudon, C., Zomahoun, H. T. V., & Croteau, J. (2017). Neuropsychological measures that predict progression from mild cognitive impairment to Alzheimer's type dementia in older adults: a systematic review and meta-analysis. Neuropsychology review, 27(4), 328-353.

Content, A., Mousty, P., & Radeau, M. (1990). Brulex. Une base de données lexicales informatisée pour le français écrit et parlé. *L'année Psychologique*, *90*(4), 551-566.

Croisile, B., Astier, J. L., & Beaumont, C. (2007). Étalonnage du test des cinq mots dans une population de sujets sains. *Revue Neurologique*, *163*(3), 323-333.

Dell’acqua, R., Lotto, L., & Job, R. (2000). Naming times and standardized norms for the Italian PD/DPSS set of 266 pictures: Direct comparisons with American, English, French, and Spanish published databases. *Behavior Research Methods, Instruments, & Computers*, *32*(4), 588-615.

Dubois, B., Touchon, J., Portet, F., Ousset, P. J., Vellas, B., & Michel, B. (2002). Les 5 mots, épreuve simple et sensible pour le diagnostic de la maladie d'Alzheimer. La presse médicale, 31(36), 1696-1699.

Frasson, P., Ghiretti, R., Catricalà, E., Pomati, S., Marcone, A., Parisi, L., ... & Clerici, F. (2011). Free and cued selective reminding test: an Italian normative study. *Neurological sciences*, *32*(6), 1057-1062.

Giovagnoli, A. R., Del Pesce, M., Mascheroni, S., Simoncelli, M., Laiacona, M., & Capitani, E. (1996). Trail making test: normative values from 287 normal adult controls. The Italian journal of neurological sciences, 17(4), 305-309.

Girtler, N., De Carli, F., Amore, M., Arnaldi, D., Bosia, L. E., Bruzzaniti, C., ... & Brugnolo, A. (2015). A normative study of the Italian printed word version of the free and cued selective reminding test. *Neurological Sciences*, *36*(7), 1127-1134.

Gollan, T. H., Weissberger, G. H., Runnqvist, E., Montoya, R. I., & Cera, C. M. (2012). Self-ratings of spoken language dominance: A Multilingual Naming Test (MINT) and preliminary norms for young and aging Spanish–English bilinguals. Bilingualism: language and cognition, 15(3), 594-615.

Goslin, J., Galluzzi, C., & Romani, C. (2014). PhonItalia: a phonological lexicon for Italian. *Behavior research methods*, *46*(3), 872-886.

Ivanova, I., Salmon, D. P., & Gollan, T. H. (2013). The multilingual naming test in Alzheimer's disease: clues to the origin of naming impairments. Journal of the International Neuropsychological Society, 19(3), 272-283.

Novelli, G., Papagno, C., Capitani, E., Laiacona, M., Vallar, G., Cappa, S.F. (1986). Tre test clinici di ricerca e produzione lessicale. Taratura su soggetti normali. Archivio di Psicologia Neurologia e Psichiatria, 4(47),477-506.

Possin, K. L., Laluz, V. R., Alcantar, O. Z., Miller, B. L., & Kramer, J. H. (2011). Distinct neuroanatomical substrates and cognitive mechanisms of figure copy performance in Alzheimer's disease and behavioral variant frontotemporal dementia. *Neuropsychologia*, *49*(1), 43-48.

Rozzini, L., Ceraso, A., Zanetti, M., Pelizzari, S., Tomasoni, E., Accardo, V., & Padovani, A. (2017). The Italian version of the five-word test: A simple diagnostic test for Dementia due to Alzheimer’s Disease in routine clinical practice. *Behavioural Neurology*, *2017*.

Stasenko, A., Jacobs, D. M., Salmon, D. P., & Gollan, T. H. (2019). The Multilingual Naming Test (MINT) as a measure of picture naming ability in Alzheimer’s disease. *Journal of the International Neuropsychological Society*, *25*(8), 821-833.

**Table 1.** Correction grids for age, education and sex and Equivalent Scores for each test (when available). To correct the raw score, the examiner has to add/subtract the values indicated on the bases of the subject age and/or education and/or sex to the raw score. Corrected score is then assigned an Equivalent Score (when available) according to the corresponding grid of values.

| test | correction grid | | | | Equivalent Scores | |
| --- | --- | --- | --- | --- | --- | --- |
|  |  |  |  |  |  |  |
| **Craft Story** |  |  |  |  |  |  |
| Immediate verbatim score | age/education | ≤8 | 9-13 | ≥14 |  |  |
|  | 40-49 | 1.795 | -0.305 | -3.455 | 0 | ≤ 4.976 |
|  | 50-59 | 2.446 | 0.346 | -2.804 | 1 | 4.977 - 7.757 |
|  | 60-69 | 3.097 | 0.997 | -2.154 | 2 | 7.758 - 10.651 |
|  | 70-79 | 3.748 | 1.648 | -1.503 | 3 | 10.652 - 13.429 |
|  | 80-89 | 4.399 | 2.298 | -0.852 | 4 | ≥ 13.430 |
|  | Corrected score= raw score - [(-0.06508) x (age - 61.30716) + 0.50004 x (education - 12.51270)] | | | | | |
|  |  |  |  |  |  |  |
| Immediate paraphrase score | education | ≤8 | 9-13 | ≥14 |  |  |
|  |  | 2.474 | 0.282 | -1.837 | 0 | ≤ 6.458 |
|  |  |  |  |  | 1 | 6.459 - 8.282 |
|  |  |  |  |  | 2 | 8.283 - 10.232 |
|  |  |  |  |  | 3 | 10.233 - 12.743 |
|  |  |  |  |  | 4 | ≥ 12.744 |
|  | Corrected score= raw score - [0.83636 x (education - 12.51270) + (-0.01767) x (education^2 - 176.63279] | | | | | |
|  |  |  |  |  |  |  |
| Recall verbatim score | age/education | ≤8 | 9-13 | ≥14 |  |  |
|  | 40-49 | 0.542 | -1.100 | -3.562 | 0 | ≤ 3.128 |
|  | 50-59 | 1.579 | -0.063 | -2.525 | 1 | 3.129 - 5.562 |
|  | 60-69 | 2.616 | 0.974 | -1.488 | 2 | 5.563 - 8.259 |
|  | 70-79 | 3.652 | 2.011 | -0.451 | 3 | 8.260 - 10.918 |
|  | 80-89 | 4.689 | 3.048 | 0.585 | 4 | ≥10.919 |
|  | Corrected score= raw score - [(-0.10368) x (age - 61.30716) + 0.39085 x (education - 12.51270)] | | | | | |
|  |  |  |  |  |  |  |
| Recall paraphrase score | age/education | ≤8 | 9-13 | ≥14 |  |  |
|  | 40-49 | 1.479 | -0.109 | -2.490 | 0 | ≤ 5.553 |
|  | 50-59 | 1.896 | 0.309 | -2.073 | 1 | 5.554 - 7.333 |
|  | 60-69 | 2.314 | 0.726 | -1.656 | 2 | 7.334 - 9.427 |
|  | 70-79 | 2.731 | 1.143 | -1.238 | 3 | 9.428 - 11.511 |
|  | 80-89 | 3.148 | 1.561 | -0.821 | 4 | ≥11.512 |
|  | Corrected score= raw score - [(-0.04173) x (age - 61.30716) + 0.37803 x (education - 12.51270)] | | | | | |
|  |  |  |  |  |  |  |
| **Five Words Test** |  |  |  |  |  |  |
| Immediate free recall | age |  |  |  |  |  |
|  | 40-49 | -0.207 |  |  | 0 | ≤ 2.831 |
|  | 50-59 | -0.080 |  |  | 1 | 2.832 - 3.212 |
|  | 60-69 | 0.047 |  |  | 2 | 3.213 - 3.933 |
|  | 70-79 | 0.174 |  |  | 3 | 3.934 - 4.338 |
|  | 80-89 | 0.300 |  |  | 4 | ≥ 4.339 |
|  | Corrected score= raw score - [(-0.01268) x (age - 61.30716)] | | | | | |
|  |  |  |  |  |  |  |
| Immediate cued recall | age |  |  |  |  |  |
|  | 40-49 | 0.204 |  |  | - | |
|  | 50-59 | 0.079 |  |  |  |  |
|  | 60-69 | -0.046 |  |  |  |  |
|  | 70-79 | -0.171 |  |  |  |  |
|  | 80-89 | -0.296 |  |  |  |  |
|  | Corrected score= raw score - [0.01251 x (age - 61.30716)] | | | | | |
|  |  |  |  |  |  |  |
| Immediate total-weighted | age |  |  |  |  |  |
|  | 40-49 | -0.209 |  |  | 0 | ≤ 7.124 |
|  | 50-59 | -0.081 |  |  | 1 | 7.125 - 8.099 |
|  | 60-69 | 0.047 |  |  | 2 | 8.100 - 8.919 |
|  | 70-79 | 0.176 |  |  | 3 | 8.920 - 9.343 |
|  | 80-89 | 0.304 |  |  | 4 | ≥ 9.344 |
|  | Corrected score= raw score - [(-0.01284) x (age - 61.30716)] | | | | | |
|  |  |  |  |  |  |  |
| Delayed free recall | age/education | ≤8 | 9-13 | ≥14 |  |  |
|  | 40-49 | -0.122 | -0.495 | -0.536 | 0 | ≤ 1.775 |
|  | 50-59 | 0.127 | -0.247 | -0.288 | 1 | 1.776 – 2.651 |
|  | 60-69 | 0.375 | 0.002 | -0.039 | 2 | 2.652 – 3.436 |
|  | 70-79 | 0.624 | 0.251 | 0.209 | 3 | 3.437 - 4.008 |
|  | 80-89 | 0.873 | 0.499 | 0.458 | 4 | ≥ 4.009 |
|  | Corrected score= raw score - [(-0.02486) x (age - 61.30716) + 0.42710 x (education - 12.51270) + (-0.02606) x (education^2 - 176.63279) + 0.000519 x (education^3 - 2706.45727)] | | | | | |
|  |  |  |  |  |  |  |
| Delayed cued recall | age/education | ≤8 | 9-13 | ≥14 |  |  |
|  | 40-49 | 0.064 | 0.195 | 0.392 | 0 | ≥ 2.472 |
|  | 50-59 | -0.084 | 0.047 | 0.243 | 1 | 2.471 – 1.894 |
|  | 60-69 | -0.233 | -0.102 | 0.094 | 2 | 1.893 – 1.146 |
|  | 70-79 | -0.382 | -0.251 | -0.054 | 3 | 1.145 - 0.693 |
|  | 80-89 | -0.530 | -0.399 | -0.203 | 4 | ≤ 0.692 |
|  | Corrected score= raw score - [(0.01486) x (age - 61.30716) + (-0.03118) x (education - 12.51270)] | | | | | |
|  |  |  |  |  |  |  |
| Delayed total recall | age/education | ≤8 | 9-13 | ≥14 |  |  |
|  | 40-49 | -0.078 | -0.245 | -0.197 | - | |
|  | 50-59 | 0.030 | -0.137 | -0.089 |  |  |
|  | 60-69 | 0.138 | -0.029 | 0.019 |  |  |
|  | 70-79 | 0.246 | 0.079 | 0.127 |  |  |
|  | 80-89 | 0.354 | 0.187 | 0.235 |  |  |
|  | Corrected score= raw score - [(-0.01081) x (age - 61.30716) + 0.23934 x (education - 12.51270) + (-0.01546) x (education^2 - 176.63279) + 0.00031 x (education^3 - 2706.45727)] | | | | | |
|  |  |  |  |  |  |  |
| Delayed total-weighted | age/education | ≤8 | 9-13 | ≥14 |  |  |
|  | 40-49 | -0.200 | -0.740 | -0.734 | 0 | ≤ 5.485 |
|  | 50-59 | 0.157 | -0.384 | -0.377 | 1 | 5.486 – 6.664 |
|  | 60-69 | 0.513 | -0.027 | -0.020 | 2 | 6.665 – 8.073 |
|  | 70-79 | 0.870 | 0.330 | 0.337 | 3 | 8.074 - 8.901 |
|  | 80-89 | 1.227 | 0.687 | 0.693 | 4 | ≥ 8.902 |
|  | Corrected score= raw score - [(-0.03567) x (age - 61.30716) + 0.66644 x (education - 12.51270) + (-0.04152) x (education^2 - 176.63279) + 0.00083 x (education^3 - 2706.45727)] | | | | | |
|  |  |  |  |  |  |  |
| Total free recall | age/education | ≤8 | 9-13 | ≥14 |  |  |
|  | 40-49 | -0.241 | -0.714 | -0.777 | 0 | ≤ 5.193 |
|  | 50-59 | 0.130 | -0.343 | -0.406 | 1 | 5.194 - 6.456 |
|  | 60-69 | 0.501 | 0.028 | -0.035 | 2 | 6.457 – 7.532 |
|  | 70-79 | 0.871 | 0.399 | 0.336 | 3 | 7.533 – 8.513 |
|  | 80-89 | 1.242 | 0.770 | 0.707 | 4 | ≥ 8.514 |
|  | Corrected score= raw score - [(-0.03708) x (age - 61.30716) + 0.52809 x (education - 12.51270) + (-0.03194) x (education^2 - 176.63279) + 0.00063 x (education^3 - 2706.45727)] | | | | | |
|  |  |  |  |  |  |  |
| Total cued recall | age/education | ≤8 | 9-13 | ≥14 |  |  |
|  | 40-49 | 0.188 | 0.378 | 0.663 | 0 | ≥ 3.833 |
|  | 50-59 | -0.086 | 0.104 | 0.389 | 1 | 3.832- 2.941 |
|  | 60-69 | -0.359 | -0.169 | 0.115 | 2 | 2.940 – 1.832 |
|  | 70-79 | -0.633 | -0.443 | -0.158 | 3 | 1.831 – 1.211 |
|  | 80-89 | -0.907 | -0.717 | -0.432 | 4 | ≤ 1.210 |
|  | Corrected score= raw score - [0.02737 x (age - 61.30716) + (-0.04521) x (education - 12.51270)] | | | | | |
|  |  |  |  |  |  |  |
| Total recall | age/education | ≤8 | 9-13 | ≥14 |  |  |
|  | 40-49 | -0.068 | -0.215 | -0.255 | - | |
|  | 50-59 | 0.049 | -0.099 | -0.139 |  |  |
|  | 60-69 | 0.165 | 0.017 | -0.023 |  |  |
|  | 70-79 | 0.281 | 0.133 | 0.094 |  |  |
|  | 80-89 | 0.397 | 0.250 | 0.210 |  |  |
|  | Corrected score= raw score - [(-0.01163) x (age - 61.30716) + 0.08411 x (education - 12.51270) + (-0.00275) x (education^2 - 176.63279)] | | | | | |
|  |  |  |  |  |  |  |
| Total-weighted recall | age |  |  |  |  |  |
|  | 40-49 | -0.786 |  |  | 0 | ≤ 13.335 |
|  | 50-59 | -0.304 |  |  | 1 | 13.336-15.503 |
|  | 60-69 | 0.178 |  |  | 2 | 15.504-17.094 |
|  | 70-79 | 0.66 |  |  | 3 | 17.095-18.455 |
|  | 80-89 | 1.143 |  |  | 4 | ≥ 18.456 |
|  | Corrected score= raw score - [(-0.04822) x (age - 61.30716)] | | | | | |
|  |  |  |  |  |  |  |
| **Picture Naming** |  |  |  |  |  |  |
| Correct without cue score | age/education | ≤8 | 9-13 | ≥14 |  |  |
|  | 40-49 | 0.760 | -0.524 | -0.862 | - | |
|  | 50-59 | 0.637 | -0.647 | -0.986 |  |  |
|  | 60-69 | 0.890 | -0.395 | -0.733 |  |  |
|  | 70-79 | 1.518 | 0.234 | -0.104 |  |  |
|  | 80-89 | 2.523 | 1.239 | 0.901 |  |  |
|  | Corrected score= raw score - [0.20046 x (age - 61.30716) + (-0.00188) x (age^2 - 3921.75520) + 1.14103 x (education - 12.51270) + (-0.06142) x (education^2 - 176.63279) + 0.00107 x (education^3 - 2706.45727)] | | | | | |
|  |  |  |  |  |  |  |
| Correct with cue score | age/education | ≤8 | 9-13 | ≥14 |  |  |
|  | 40-49 | 0.018 | 0.080 | 0.173 | - | |
|  | 50-59 | -0.045 | 0.017 | 0.110 |  |  |
|  | 60-69 | -0.108 | -0.046 | 0.048 |  |  |
|  | 70-79 | -0.170 | -0.108 | -0.015 |  |  |
|  | 80-89 | -0.233 | -0.171 | -0.078 |  |  |
|  | Corrected score= raw score - [(0.00627) x (age - 61.30716) + (-0.01479) x (education - 12.51270)] | | | | | |
|  |  |  |  |  |  |  |
| Correct total score | age/education | ≤8 | 9-13 | ≥14 |  |  |
|  | 40-49 | 0.741 | -0.457 | -0.724 | - | |
|  | 50-59 | 0.604 | -0.594 | -0.861 |  |  |
|  | 60-69 | 0.805 | -0.393 | -0.660 |  |  |
|  | 70-79 | 1.344 | 0.146 | -0.121 |  |  |
|  | 80-89 | 2.221 | 1.023 | 0.756 |  |  |
|  | Corrected score= raw score - [0.18245 x (age - 61.30716) + (-0.00169) x (age^2 - 3921.75520) + 1.09496 x (education - 12.51270) + (-0.05963) x (education^2 - 176.63279) + 0.00104 x (education^3 - 2706.45727)] | | | | | |
|  |  |  |  |  |  |  |
| **Semantic Fluency** |  |  |  |  |  |  |
| Animals correct score (< 30 s) | age/education | ≤8 | 9-13 | ≥14 |  |  |
|  | 40-49 | 0.538 | -0.987 | -2.003 | 0 | ≤ 6.532 |
|  | 50-59 | 1.378 | -0.147 | -1.163 | 1 | 6.533-9.154 |
|  | 60-69 | 2.218 | 0.694 | -0.323 | 2 | 9.155-11.112 |
|  | 70-79 | 3.058 | 1.534 | 0.518 | 3 | 11.113-13.401 |
|  | 80-89 | 3.898 | 2.374 | 1.358 | 4 | ≥ 13.402 |
|  | Corrected score= raw score - [(-0.084095) x (age - 61.30716) + 0.254145 x (education - 12.51270)] | | | | | |
|  |  |  |  |  |  |  |
| Animals correct score (> 30 s) | age/education | ≤8 | 9-13 | ≥14 |  |  |
|  | 40-49 | 1.801 | -0.384 | -1.273 | 0 | ≤ 0.164 |
|  | 50-59 | 2.101 | -0.084 | -0.973 | 1 | 0.165-2.715 |
|  | 60-69 | 2.400 | 0.216 | -0.673 | 2 | 2.716-4.985 |
|  | 70-79 | 2.700 | 0.516 | -0.373 | 3 | 4.986-6.940 |
|  | 80-89 | 3.000 | 0.816 | -0.074 | 4 | ≥ 6.941 |
|  | Corrected score= raw score - [(-0.029983) x (age - 61.30716) + 0.590736 x (education - 12.51270) + (-0.014168) x (education^2 - 176.63279)] | | | | | |
|  |  |  |  |  |  |  |
| Animals total correct score (60 s) | age/education | ≤8 | 9-13 | ≥14 |  |  |
|  | 40-49 | 2.834 | -1.566 | -3.414 | 0 | ≤ 10.177 |
|  | 50-59 | 3.952 | -0.449 | -2.297 | 1 | 10.178-13.719 |
|  | 60-69 | 5.069 | 0.669 | -1.179 | 2 | 13.720-17.066 |
|  | 70-79 | 6.187 | 1.787 | -0.061 | 3 | 17.067-20.408 |
|  | 80-89 | 7.305 | 2.905 | 1.057 | 4 | ≥ 20.409 |
|  | Corrected score= raw score - [(-0.111792) x (age - 61.30716) + 1.167452 x (education - 12.51270) + (-0.027132) x (education^2 - 176.63279)] | | | | | |
|  |  |  |  |  |  |  |
| Animals violations | age |  |  |  |  |  |
|  | 40-49 | -0.571 |  |  | 0 | ≥ 2.846 |
|  | 50-59 | -0.221 |  |  | 1 | 2.845-1.386 |
|  | 60-69 | 0.129 |  |  | 2 | 1.385-0.776 |
|  | 70-79 | 0.479 |  |  | 3 | 0.775-0.344 |
|  | 80-89 | 0.829 |  |  | 4 | ≤ 0.343 |
|  | Corrected score= raw score - [(-0.035477) x (age - 61.30716)] | | | | | |
|  |  |  |  |  |  |  |
| Vegetables correct score (< 30 s) | Female, age/education | ≤8 | 9-13 | ≥14 |  |  |
|  | 40-49 | -1.719 | -2.536 | -3.081 | 0 | ≤ 3.138 |
|  | 50-59 | -1.221 | -2.039 | -2.584 | 1 | 3.139-4.652 |
|  | 60-69 | -0.724 | -1.542 | -2.087 | 2 | 4.653-6.441 |
|  | 70-79 | -0.227 | -1.044 | -1.589 | 3 | 6.442-8.208 |
|  | 80-89 | 0.271 | -0.547 | -1.092 | 4 | ≥ 8.209 |
|  | Male, age/education | ≤8 | 9-13 | ≥14 |  |  |
|  | 40-49 | 0.212 | -0.605 | -1.150 | 0 | ≤ 3.138 |
|  | 50-59 | 0.710 | -0.108 | -0.653 | 1 | 3.139-4.652 |
|  | 60-69 | 1.207 | 0.390 | -0.155 | 2 | 4.653-6.441 |
|  | 70-79 | 1.705 | 0.887 | 0.342 | 3 | 6.442-8.208 |
|  | 80-89 | 2.202 | 1.385 | 0.840 | 4 | ≥ 8.209 |
|  | Corrected score= raw score - [(-0.04974) x (age - 61.30716) + 0.13625 x (education - 12.51270)] - 1.93135 (if female) + 1.93135 (if male) | | | | | |
|  |  |  |  |  |  |  |
| Vegetables correct score (> 30 s) | Female, education | ≤8 | 9-13 | ≥14 |  |  |
|  |  | -0.143 | -0.490 | -0.721 | 0 | 0 |
|  |  |  |  |  | 1 | 0- 0.394 |
|  |  |  |  |  | 2 | 0.395-1.510 |
|  |  |  |  |  | 3 | 1.511-3.106 |
|  |  |  |  |  | 4 | ≥ 3.107 |
|  | Male, education | ≤8 | 9-13 | ≥14 | 0 | 0 |
|  |  | 0.434 | 0.087 | -0.144 | 1 | 0- 0.394 |
|  |  |  |  |  | 2 | 0.395-1.510 |
|  |  |  |  |  | 3 | 1.511-3.106 |
|  |  |  |  |  | 4 | ≥ 3.107 |
|  | Corrected score= raw score - [0.05777 x (education - 12.51270)] - 0.57742 (if female) + 0.57742 (if male) | | | | | |
|  |  |  |  |  |  |  |
| Vegetables total correct score (60 s) | Female, age/education | ≤8 | 9-13 | ≥14 |  |  |
|  | 40-49 | -1.994 | -3.130 | -3.888 | 0 | ≤ 4.506 |
|  | 50-59 | -1.440 | -2.577 | -3.334 | 1 | 4.507-6.924 |
|  | 60-69 | -0.887 | -2.023 | -2.781 | 2 | 6.925-9.102 |
|  | 70-79 | -0.333 | -1.470 | -2.227 | 3 | 9.103-11.177 |
|  | 80-89 | 0.220 | -0.916 | -1.674 | 4 | ≥ 11.178 |
|  | Male, age/education | ≤8 | 9-13 | ≥14 |  |  |
|  | 40-49 | 0.520 | -0.616 | -1.374 | 0 | ≤ 4.506 |
|  | 50-59 | 1.074 | -0.063 | -0.820 | 1 | 4.507-6.924 |
|  | 60-69 | 1.627 | 0.491 | -0.267 | 2 | 6.925-9.102 |
|  | 70-79 | 2.181 | 1.044 | 0.287 | 3 | 9.103-11.177 |
|  | 80-89 | 2.734 | 1.598 | 0.840 | 4 | ≥ 11.178 |
|  | Corrected score= raw score - [(-0.05535) x (age - 61.30716) +0.18940 x (education - 12.51270)] - 2.51409 (if female) + 2.51409 (if male) | | | | | |
|  |  |  |  |  |  |  |
| Vegetables perseverations | Females | -0.238 |  |  | - | |
|  | Corrected score= raw score + 0.23826 (if female) - 0.23826 (if male) | | | | | |
|  |  |  |  |  |  |  |
| Total correct score (60 s) | Female, age/education | ≤8 | 9-13 | ≥14 |  |  |
|  | 40-49 | 0.476 | -5.331 | -7.934 | 0 | ≤ 16.990 |
|  | 50-59 | 2.148 | -3.660 | -6.262 | 1 | 16.991-21.627 |
|  | 60-69 | 3.820 | -1.988 | -4.590 | 2 | 21.628-26.493 |
|  | 70-79 | 5.492 | -0.316 | -2.918 | 3 | 26.494-31.415 |
|  | 80-89 | 7.164 | 1.356 | -1.246 | 4 | ≥ 31.416 |
|  | Male, age/education | ≤8 | 9-13 | ≥14 |  |  |
|  | 40-49 | 3.548 | -2.260 | -4.862 | 0 | ≤ 16.990 |
|  | 50-59 | 5.220 | -0.588 | -3.190 | 1 | 16.991-21.627 |
|  | 60-69 | 6.892 | 1.084 | -1.518 | 2 | 21.628-26.493 |
|  | 70-79 | 8.564 | 2.756 | 0.154 | 3 | 26.494-31.415 |
|  | 80-89 | 10.236 | 4.428 | 1.826 | 4 | ≥ 31.416 |
|  | Corrected score= raw score - [(-0.167194) x (age - 61.30716) + 1.475677 x (education - 12.51270) + (-0.031735) x (education^2 - 176.63279)] -3.071774 (if female) + 3.071774 (if male) | | | | | |
|  |  |  |  |  |  |  |
| Total violations | age |  |  |  |  |  |
|  | 40-49 | -0.560 |  |  | 0 | ≥ 10.715 |
|  | 50-59 | -0.217 |  |  | 1 | 10.714-3.612 |
|  | 60-69 | 0.127 |  |  | 2 | 3.611-1.677 |
|  | 70-79 | 0.470 |  |  | 3 | 1.676-0.715 |
|  | 80-89 | 0.814 |  |  | 4 | ≤ 0.714 |
|  | Corrected score= raw score - [(-0.0343532) x (age - 61.30716)] | | | | | |
|  |  |  |  |  |  |  |
| **Phonemic Fluency** |  |  |  |  |  |  |
| Letter F correct score (< 30 s) | Female, age/education | ≤8 | 9-13 | ≥14 |  |  |
|  | 40-49 | 0.573 | -1.823 | -2.984 | 0 | ≤ 3.746 |
|  | 50-59 | 1.217 | -1.179 | -2.340 | 1 | 3.747-4.950 |
|  | 60-69 | 1.861 | -0.535 | -1.696 | 2 | 4.951-6.454 |
|  | 70-79 | 2.505 | 0.109 | -1.052 | 3 | 6.455-7.874 |
|  | 80-89 | 3.150 | 0.753 | -0.408 | 4 | ≥ 7.875 |
|  | Male, age/education | ≤8 | 9-13 | ≥14 |  |  |
|  | 40-49 | 1.607 | -0.789 | -1.950 | 0 | ≤ 3.746 |
|  | 50-59 | 2.251 | -0.145 | -1.306 | 1 | 3.747-4.950 |
|  | 60-69 | 2.895 | 0.499 | -0.662 | 2 | 4.951-6.454 |
|  | 70-79 | 3.539 | 1.143 | -0.018 | 3 | 6.455-7.874 |
|  | 80-89 | 4.183 | 1.787 | 0.626 | 4 | ≥ 7.875 |
|  | Corrected score= raw score - [(-0.06441) x (age - 61.30716) + 0.57410 x (education - 12.51270) + (-0.01092) x (education^2 - 176.63279)] -1.03377 (if female) + 1.03377 (if male) | | | | | |
|  |  |  |  |  |  |  |
| Letter F correct score (> 30 s) | age/education | ≤8 | 9-13 | ≥14 |  |  |
|  | 40-49 | 1.236 | -0.108 | -1.004 | 0 | ≤ 0.718 |
|  | 50-59 | 1.510 | 0.166 | -0.730 | 1 | 0.719-1.976 |
|  | 60-69 | 1.784 | 0.440 | -0.456 | 2 | 1.977-3.467 |
|  | 70-79 | 2.058 | 0.714 | -0.182 | 3 | 3.468-5.052 |
|  | 80-89 | 2.332 | 0.988 | 0.092 | 4 | ≥ 5.053 |
|  | Corrected score= raw score - [(-0.02740) x (age - 61.30716) + 0.22400 x (education - 12.51270)] | | | | | |
|  |  |  |  |  |  |  |
| Letter F total correct score (60 s) | age/education | ≤8 | 9-13 | ≥14 |  |  |
|  | 40-49 | 2.402 | -0.698 | -2.764 | 0 | ≤ 6.747 |
|  | 50-59 | 3.309 | 0.209 | -1.857 | 1 | 6.748-8.718 |
|  | 60-69 | 4.216 | 1.116 | -0.950 | 2 | 8.719-11.094 |
|  | 70-79 | 5.123 | 2.023 | -0.043 | 3 | 11.095-13.720 |
|  | 80-89 | 6.030 | 2.930 | 0.864 | 4 | ≥ 13.721 |
|  | Corrected score= raw score - [(-0.09067) x (age - 61.30716) + 0.51662 x (education - 12.51270)] | | | | | |
|  |  |  |  |  |  |  |
| Letter L correct score (< 30 s) | Female, age/education | ≤8 | 9-13 | ≥14 |  |  |
|  | 40-49 | 0.385 | -1.328 | -2.470 | 0 | ≤ 2.346 |
|  | 50-59 | 0.815 | -0.897 | -2.039 | 1 | 2.347-3.614 |
|  | 60-69 | 1.246 | -0.467 | -1.609 | 2 | 3.615-4.974 |
|  | 70-79 | 1.676 | -0.036 | -1.178 | 3 | 4.975-6.492 |
|  | 80-89 | 2.107 | 0.394 | -0.747 | 4 | ≥ 6.493 |
|  | Male, age/education | ≤8 | 9-13 | ≥14 |  |  |
|  | 40-49 | 1.442 | -0.270 | -1.412 | 0 | ≤ 2.346 |
|  | 50-59 | 1.873 | 0.160 | -0.982 | 1 | 2.347-3.614 |
|  | 60-69 | 2.303 | 0.591 | -0.551 | 2 | 3.615-4.974 |
|  | 70-79 | 2.734 | 1.021 | -0.120 | 3 | 4.975-6.492 |
|  | 80-89 | 3.164 | 1.452 | 0.310 | 4 | ≥ 6.493 |
|  | Corrected score= raw score - [(-0.04306) x (age - 61.30716) + 0.28543 x (education - 12.51270)] -1.05767 (if female) + 1.05767 (if male) | | | | | |
|  |  |  |  |  |  |  |
| Letter L correct score (> 30 s) | age/education | ≤8 | 9-13 | ≥14 |  |  |
|  | 40-49 | 1.236 | -0.108 | -1.004 | 0 | 0 |
|  | 50-59 | 1.510 | 0.166 | -0.730 | 1 | 0-1.284 |
|  | 60-69 | 1.784 | 0.440 | -0.456 | 2 | 1.285-2.424 |
|  | 70-79 | 2.058 | 0.714 | -0.182 | 3 | 2.425-3.714 |
|  | 80-89 | 2.332 | 0.988 | 0.092 | 4 | ≥ 3.715 |
|  | Corrected score= raw score - [(-0.02485) x (age - 61.30716) + 0.196605 x (education - 12.51270)] | | | | | |
|  |  |  |  |  |  |  |
| Letter L total correct score (60 s) | Female, age/education | ≤8 | 9-13 | ≥14 |  |  |
|  | 40-49 | 2.597 | -0.309 | -2.246 | 0 | ≤ 3.520 |
|  | 50-59 | 3.277 | 0.372 | -1.566 | 1 | 3.521-5.581 |
|  | 60-69 | 3.958 | 1.052 | -0.885 | 2 | 5.582-7.916 |
|  | 70-79 | 4.638 | 1.732 | -0.205 | 3 | 7.917-10.112 |
|  | 80-89 | 5.318 | 2.412 | 0.475 | 4 | ≥ 10.113 |
|  | Male, age/education | ≤8 | 9-13 | ≥14 |  |  |
|  | 40-49 | 2.529 | -0.377 | -2.314 | 0 | ≤ 3.520 |
|  | 50-59 | 3.209 | 0.304 | -1.634 | 1 | 3.521-5.581 |
|  | 60-69 | 3.890 | 0.984 | -0.953 | 2 | 5.582-7.916 |
|  | 70-79 | 4.570 | 1.664 | -0.273 | 3 | 7.917-10.112 |
|  | 80-89 | 5.250 | 2.344 | 0.407 | 4 | ≥ 10.113 |
|  | Corrected score= raw score - [(-0.06802) x (age - 61.30716) + 0.48430 x (education - 12.51270)] -1.42726 (if female) + 1.42726 (if male) | | | | | |
|  |  |  |  |  |  |  |
| Total correct score (60 s) | age/education | ≤8 | 9-13 | ≥14 |  |  |
|  | 40-49 | 5.810 | -1.415 | -5.135 | 0 | ≤ 10.888 |
|  | 50-59 | 7.316 | 0.092 | -3.628 | 1 | 10.889-15.505 |
|  | 60-69 | 8.822 | 1.598 | -2.122 | 2 | 15.506-18.954 |
|  | 70-79 | 10.329 | 3.104 | -0.615 | 3 | 18.955-24.695 |
|  | 80-89 | 11.835 | 4.611 | 0.891 | 4 | ≥ 24.696 |
|  | Corrected score= raw score - [(-0.150638) x (age - 61.30716) + 6.68580 x (√education - 3.47242)] | | | | | |
|  |  |  |  |  |  |  |
| Total perseverations | education | ≤8 | 9-13 | ≥14 |  |  |
|  |  | 0.308 | 0.062 | -0.102 | 0 | ≥ 3.980 |
|  |  |  |  |  | 1 | 3.979-2.775 |
|  |  |  |  |  | 2 | 2.774-1.185 |
|  |  |  |  |  | 3 | 1.184-0.775 |
|  |  |  |  |  | 4 | ≤ 0.774 |
|  | Corrected score= raw score - [0.04101 x (education - 12.51270)] | | | | | |
|  |  |  |  |  |  |  |
| **Benson Figure** |  |  |  |  |  |  |
| Copy | education | ≤8 | 9-13 | ≥14 |  |  |
|  |  | 1.251 | 0.157 | -0.064 | 0 | ≤ 11.931 |
|  |  |  |  |  | 1 | 11.932 - 12.969 |
|  |  |  |  |  | 2 | 12.970 - 14.819 |
|  |  |  |  |  | 3 | 14.820 - 15.819 |
|  |  |  |  |  | 4 | ≥ 15.820 |
|  | Corrected score= raw score - [1.12351 x (education - 12.51270) + (-0.06555) x (education^2 - 176.63279) + 0.00126 x (education^3 - 2706.45727)] | | | | | |
|  |  |  |  |  |  |  |
| Recall | age/education | ≤8 | 9-13 | ≥14 |  |  |
|  | 40-49 | 0.158 | -1.075 | -1.820 | 0 | ≤ 5.481 |
|  | 50-59 | 1.002 | -0.232 | -0.976 | 1 | 5.482- 7.607 |
|  | 60-69 | 1.845 | 0.611 | -0.133 | 2 | 7.608 - 9.725 |
|  | 70-79 | 2.688 | 1.454 | 0.710 | 3 | 9.753 - 11.493 |
|  | 80-89 | 3.531 | 2.297 | 1.553 | 4 | ≥ 11.494 |
|  | Corrected score= raw score - [(-0.08431) x (age - 61.30716) + 0.59126 x (education - 12.51270) + (-0.01672) x (education^2 - 176.63279)] | | | | | |
|  |  |  |  |  |  |  |
| **Digit Span Forward** |  |  |  |  |  |  |
| Number of correct trials | Female, age/education | ≤8 | 9-13 | ≥14 |  |  |
|  | 40-49 | 1.010 | 0.156 | -0.674 | 0 | ≤ 3.519 |
|  | 50-59 | 1.278 | 0.425 | -0.405 | 1 | 3.520 - 4.434 |
|  | 60-69 | 1.547 | 0.694 | -0.136 | 2 | 4.435 - 5.442 |
|  | 70-79 | 1.816 | 0.962 | 0.133 | 3 | 5.443 - 6.469 |
|  | 80-89 | 2.084 | 1.231 | 0.401 | 4 | ≥ 6.470 |
|  | Male, age/education | ≤8 | 9-13 | ≥14 |  |  |
|  | 40-49 | 0.318 | -0.535 | -1.365 | 0 | ≤ 3.519 |
|  | 50-59 | 0.587 | -0.267 | -1.096 | 1 | 3.520 - 4.434 |
|  | 60-69 | 0.855 | 0.002 | -0.828 | 2 | 4.435 - 5.442 |
|  | 70-79 | 1.124 | 0.271 | -0.559 | 3 | 5.443 - 6.469 |
|  | 80-89 | 1.393 | 0.539 | -0.290 | 4 | ≥ 6.470 |
|  | Corrected score= raw score - [(-0.02687) x (age - 61.30716) + 0.32443 x (education - 12.51270) + (-0.00681) x (education^2 - 176.63279)] - 0.34579 (if female) + 0.34579 (if male) | | | | | |
|  |  |  |  |  |  |  |
| Span length | age/education | ≤8 | 9-13 | ≥14 |  |  |
|  | 40-49 | 0.192 | -0.217 | -0.581 | 0 | ≤ 3.917 |
|  | 50-59 | 0.512 | 0.103 | -0.261 | 1 | 3.918 - 4.672 |
|  | 60-69 | 0.495 | 0.086 | -0.278 | 2 | 4.678 - 4.967 |
|  | 70-79 | 0.411 | 0.002 | -0.362 | 3 | 4.968 – 5.724 |
|  | 80-89 | 0.530 | 0.121 | -0.243 | 4 | ≥ 5.725 |
|  | Corrected score= raw score - [(-0.60450) x (age - 61.30716) + 0.00911 x (age^2 - 3921.75520) + (-0.00005) x (age^3 -260837.49192) + 0.16450 x (education - 12.51270) + (-0.00377) x (education^2 - 176.63279)] | | | | | |
|  |  |  |  |  |  |  |
| **Digit Span Backward** |  |  |  |  |  |  |
| Number of correct trials | age/education | ≤8 | 9-13 | ≥14 |  |  |
|  | 40-49 | 0.131 | -0.677 | -1.373 | 0 | ≤ 3.023 |
|  | 50-59 | 0.752 | -0.055 | -0.752 | 1 | 3.024 - 3.819 |
|  | 60-69 | 1.153 | 0.346 | -0.350 | 2 | 3.820 - 4.760 |
|  | 70-79 | 1.335 | 0.527 | -0.169 | 3 | 4.761 - 5.591 |
|  | 80-89 | 1.296 | 0.488 | -0.208 | 4 | ≥ 5.592 |
|  | Corrected score= raw score - [(-0.17223) x (age - 61.30716) + 0.00110 x (age^2 - 3921.75520) + 0.33063 x (education - 12.51270) + (-0.00778) x (education^2 - 176.63279)] | | | | | |
|  |  |  |  |  |  |  |
| Span length | age/education | ≤8 | 9-13 | ≥14 |  |  |
|  | 40-49 | -0.035 | -0.385 | -0.801 | 0 | ≤ 2.751 |
|  | 50-59 | 0.302 | -0.048 | -0.464 | 1 | 2.752- 3.038 |
|  | 60-69 | 0.531 | 0.181 | -0.235 | 2 | 3.039- 3.666 |
|  | 70-79 | 0.653 | 0.303 | -0.113 | 3 | 3.667- 4.134 |
|  | 80-89 | 0.667 | 0.317 | -0.099 | 4 | ≥ 4.135 |
|  | Corrected score= raw score - [(-0.08738) x (age - 61.30716) + 0.00054 x (age^2 - 3921.75520) + 0.49369 x (√education - 3.47242)] | | | | | |
|  |  |  |  |  |  |  |
| **Trial Making Test** |  |  |  |  |  |  |
| Part A (seconds) | age/education | ≤8 | 9-13 | ≥14 |  |  |
|  | 40-49 | -14.449 | 13.313 | 17.171 | 0 | ≥ 93.699 |
|  | 50-59 | -20.109 | 7.653 | 11.511 | 1 | 67.446-93.698 |
|  | 60-69 | -23.392 | 4.371 | 8.229 | 2 | 53.992-67.445 |
|  | 70-79 | -31.875 | -4.113 | -0.255 | 3 | 44.570-53.991 |
|  | 80-89 | -53.138 | -25.375 | -21.517 | 4 | ≤ 44.569 |
|  | Corrected score= raw score - [13.0899 x (age - 61.30716) + (-0.22028) x (age^2 - 3921.75510) + 0.00126 x (age^3 -260837.49192) + (-14.0951) x (education - 12.51270) + 0.74539 x (education^2 - 176.63279) + (-0.01223) x (education^3 - 2706.45727)] | | | | | |
|  |  |  |  |  |  |  |
| Part B (seconds) | age/education | ≤8 | 9-13 | ≥14 |  |  |
|  | 40-49 | -39.754 | 31.761 | 45.819 | 0 | ≥ 226.340 |
|  | 50-59 | -46.886 | 24.629 | 38.687 | 1 | 171.883-226.339 |
|  | 60-69 | -64.961 | 6.554 | 20.613 | 2 | 142.029-171.882 |
|  | 70-79 | -93.979 | -22.464 | -8.406 | 3 | 113.790-142.028 |
|  | 80-89 | -133.939 | -62.425 | -48.367 | 4 | ≤ 113.789 |
|  | Corrected score= raw score - [(-4.75843) x (age - 61.30716) + 0.05472 x (age^2 - 3921.75520) + (-33.9067) x (education - 12.51270) + 1.73791 x (education^2 - 176.63279) + (-0.02895) x (education^3 - 2706.45727)] | | | | | |
|  |  |  |  |  |  |  |
| Part B-A (seconds) | age/education | ≤8 | 9-13 | ≥14 |  |  |
|  | 40-49 | -18.052 | 19.347 | 32.560 | 0 | ≥ 177.600 |
|  | 50-59 | -27.445 | 9.954 | 23.168 | 1 | 114.394- 177.599 |
|  | 60-69 | -39.207 | -1.808 | 11.405 | 2 | 89.025-114.393 |
|  | 70-79 | -54.956 | -17.557 | -4.344 | 3 | 67.323-88.024 |
|  | 80-89 | -78.865 | -41.466 | -28.253 | 4 | ≤ 67.322 |
|  | Corrected score= raw score - [(-46.80513) x (ln(30-age) - 3.5893) + (-10.92087) x (education - 12.51270) + 0.29298 x (education^2 - 176.63279)] | | | | | |
